# The influence of ESG practices on domestic value-added to exports during the period of technological change

**DOI:** 10.1371/journal.pone.0303248

**Published:** 2024-07-05

**Authors:** Sun Yue, BingXiang Li

**Affiliations:** 1 Department of Management Sciences and Economics, Xi’an University of Technology, Xi’an, China; 2 Director of Teaching Development Center, Director of Higher Education Research Institute of Xi’an University of Technolog, Department of Management Sciences and Economics, Xi’an University of Technology, Xi’an, China; University of Almeria: Universidad de Almeria, SPAIN

## Abstract

Sustainable development and raising the domestic value-added rate of exports (DVARE) have become essential priorities in the pursuit of high-quality economic growth. An econometric spatial model is developed in this research using data on Chinese enterprises spanning 2008 to 2019. According to a study, exports’ domestic value-added rate (DVAR) can be successfully increased using environmental, social, and governance (ESG) practices. Spatial spillover analysis demonstrates that adopting ESG practices boosts export DVAR both within and between regions. According to heterogeneity analysis, the sample’s overall increase in DVARE as a result of ESG practices is mostly attributable to the mix and processing trade organizations, the eastern area, and large firms. An examination of the underlying mechanisms shows that businesses that implement advanced technologies are able to reinforce the favorable impact of ESG practices on DVARE. This article gives evidence from real-world studies that show how ESG practices help boost Chinese exports and advance sustainable development. The findings hold significant implications for other developing nations as they make the transition towards a pattern of economic growth.

## 1. Introduction

Over the last two decades, China, the most rapidly developing country globally, has experienced an extraordinary surge in its export figures, owning a substantial achievement in economic progress. By 2020, manufactured goods had accounted for more than 90% of China’s total export value. In comparison, Current research suggests that vertical specialization, a driving force behind China’s export boom, leads to relatively little DVARE [1]. However, China’s increasing energy consumption and pollution levels, coupled with its massive exports, pose a severe problem [2]. Determining how to increase DVARE in order to achieve high-quality and sustainable development and to improve its standing internationally has emerged as one of China’s main challenges in the new era.

Since the beginning of the twenty-first century, China has made an effort to enhance its competitiveness in international trade, emphasizing the quality of its products more than just the quantity. Being the largest trader in the world, China’s social responsibility crises regularly have an impact on Chinese enterprises in international trade. Export-focused firms are frequently hindered by unseen obstacles, including continued environmental protection, completed safety inspections, and consumer rights protection [3, 4]. In this context, it is imperative to acknowledge the significance of sustainable development, which requires businesses to develop and implement management strategies and tools to meet development objectives related to social, ecological, and governance issues commonly referred to as ESG. Recognizing ESG is important for readers in specialized and non-specialized fields because it puts its significance in corporate operations and societal growth [5, 6]. The fundamental idea behind ESG is that companies must consider the concerns and priorities of key stakeholders, including the environment, society, and the public while doing trade and making investment decisions in order to achieve both sustainable growth and financial benefits [7, 8]. Given the interdependence between ESG and increasing exports, an intriguing topic is whether attempts to improve ESG practices contribute to boosting the DVAR of Chinese exports.

The aforementioned question holds considerable importance on a worldwide scale, as it pertains not only to the progress of China but also to the potential for other developing nations achieve a win-win scenario for trade and sustainable development. Many developing countries have noticed an increase in their involvement in the global market since 2000 as a result of their specialization in particular production phases [9]. Exportation plays a crucial role in the participation of Global Value Chains, as it contributes to the advancement of undeveloped countries and promotes economic expansion. Therefore, establishing international trade emerges as a crucial factor for developing countries grappling with severe environmental deterioration, social challenges, and the immense demands of economic development. However, it is noteworthy that the relationship between export upgrading and ESG practices has not received enough attention in developing countries. This study evaluates the aforementioned issues using export data and the ESG index from the China Customs and Wind database. The objective is to explore the influence of ESG practices on the process of Chinese export upgrading, specifically focusing on the exports’ DVAR.

The extant body of literature about our research largely emphases on the influence of ESG performance on investment decisions [10] market value [11], and capital cost [12, 13]. These studies consistently demonstrate that ESG performance yields favorable economic outcomes. Nevertheless, most research endeavors have been concentrated on internal matters within firms, with limited attention given to the extension of business boundaries. Current research mainly works on computing DVAR and studying the factors that affect it. The input-output (I-O) tables serve as the primary basis for measuring DVAR at the macro level [14, 15]. Several previous studies have examined the determinants of DVAR by considering several factors, such as investment in research and development trade liberalization, foreign investment, productivity, and environmental regulations [16–18]. However, studies examining the connection between ESG practices and exports DVAR is particularly lacking. Therefore, it is very important to find out if the new competitive benefits that ESG practices create can help businesses increase their domestic value-added rate of exports. This can add depth to theories of ESG and global trade.

As the digital technology wave continues to rise, more and more researchers are examining how the expansion of digital technologies affects global trade. Some academics argue that the use of advanced technology encourages export and sustainability development because it removes geographical barriers [19, 20]. In a recent survey conducted by BlackRock, 53% of respondents expressed concern over the lack of access to ESG data, which is essential for evaluating a company’s commitment to sustainability. Saxena et al argued this barrier can be removed by putting advanced technologies into practice [21]. Based on this, the study further examines whether firms adopting technological turbulence have a more pronounced relationship between ESG performance and DVARE. Furthermore, it is evident that China’s process of industrialization and urbanization is accompanied by a noticeable geographical agglomeration phenomenon, as highlighted by Zheng et al. [22]. Does China’s export trade development exhibit a spatial correlation?

This paper presents several notable contributions. Firstly, it is the original study that examines the influence of ESG practices on DVARE. Existing research largely focuses on determining how various ESG policies affect export performance. The primary research perspectives now revolve around enhancing export quality, competitiveness and comparative advantages [23, 24]. This study comprehensively investigates the impact of ESG practices on the firm DVARE. Second, in this study, we examine the potential spatial spillover effects that arise from the implementation of ESG practices on DVARE. Moreover, the study also examines the heterogeneity analysis of ESG practices. The heterogeneity impact of ESG is seen at the region and firm levels. Lastly, an examination of the underlying mechanisms reveals that the benefits of ESG practices in increasing DVARE are reinforced by technological turbulence.

The remaining studies are arranged in the following manner: The study develops hypotheses in Section 2. Data sources, ESG index construction, DVARE calculation, and econometric models are all detailed in Section 3. Section 4 comprises the presentation of the comprehensive findings. In the fifth section, a summary of the findings is presented.

## 2. Literature review and hypotheses

Given the growing significance of sustainability-related societal issues, including wealth inequality and climate change, more investors and consumers are concentrating on sustainable development [25–27]. As a result, scholars have focused more and more on how non-economic factors like environmental regulation, companies’ governance concerns and social responsibility affect corporations’ export policies. On the one hand, conventional wisdom claims that strong environmental rules raise the costs of compliance and lower enterprises’ initial investments in production, lowering their level of competition in the international market [28, 29]. Whereas, several studies argue that environmental regulations could potentially drive businesses to engage in innovation, thereby enhancing productivity and providing them with a competitive edge in the market. [30]. The continual expansion of the worldwide division of labor and the ever-more globalized economy has led to an increasing number of export items requiring the completion of intermediary items imported from other countries or regions [31]. Despite the historical tendency of firms to prioritize profitability over ESG considerations, there has been a notable increase in the significance of ESG measurements over the last few years [32, 33] The study conducted by Wu et al. indicates a significant positive correlation between the export intensity and corporate ESG performance. exhibited by firms [34]. However, it is not possible to calculate a country’s true export earnings just by monitoring its total export volume. The DVARE has increasingly been used as a benchmark for determining true income. The connection between ESG practices and DVARE is still not well discussed. The study’s first hypothesis was formed based on the discussion above.

H1: Good ESG practices of firms positively affect the domestic value-added rate of exports.

Factors such as a company’s age, size, capital stock, quality of foreign products, capital share, and sunk cost in exporting all have an impact on the company’s export intensity [35]. Employing ESG practices makes it easier for nearby cities to improve their clean and sustainable output. This approach aims to optimize the comprehensive utilization of resources, ensure efficient utilization of governance factors, minimize pollutant emissions, foster sustainable social development, and effectively control environmental pollution [33, 36]. Will there be an impact on local export trade? It is apparent that a notable geographical agglomeration phenomenon is occurring concurrently with China’s process of urbanization and industrialization [22, 37]. Based on the study conducted by Ma et al., artificial intelligence has made it possible to overcome geographical and spatial limitations, allowing for better communication and interaction across cities and regions and removing previous barriers to economic activity. [38, 39]. Hence, it is possible that geographical heterogeneity could potentially affect the extent to which ESG practice stimulates DVARE. As a result of ESG practices, neighboring cities are better able to use resources holistically, regulate pollution by reducing emissions, and strengthen clean and sustainable production. Therefore, the study hypothesis that:

H2: Spatial effects of ESG practices may be noticed on export DVAR in nearby regions.

In 2016, a study made the case that using blockchain technology to build automated, transparent, and flexible data collection methods can improve the efficiency of processing and packaging ESG reports [40]. Wu et al. present a novel architectural framework for an advanced ESG reporting system [34]. This framework enables corporate crowd-sensing of data related to the environment through the blockchain technology and Internet of Things. This platform aims to improve the ESG reporting process’s security, reliability, and transparency. The UNCTAD (United Nations Conference on Trade and Development) states that businesses face both strategic opportunities and risks as a result of rapid technological change [41].

Information and communication technology have emerged as crucial determinants of economic conduct in contemporary society. The export trade market’s participants and business scope have expanded beyond its initial geographic restrictions as a result of the rise of technological advancement [42]. Therefore, it is anticipated that companies operating in turbulent settings will demonstrate enhanced proficiency in implementing their ESG practices, thereby leading to increased export DVAR. Consequently, we propose a hypothesis:

H3: The correlation between ESG practices and exports DVAR is strengthened with higher degrees of technical turbulence.

## 3. Methodology and variable selection

### 3.1 The empirical model

In order to conduct a statistical analysis between ESG practices and firm DVARE, the study established the subsequent regression model.


$$DVARE_{it}=\alpha_{0}+\alpha_{1}*ESG+\alpha_{2}*X+\alpha_{3}*Z+\theta_{j}+\gamma_{t}+\varepsilon_{it}$$ (1)

The letter X represents a group of control variables that change during the course of time within an organization i.e. enterprise age, enterprise size, enterprise type (foe),export density (exd), ownership property (soe), dummy of exit (exit),dummy of trade pattern(trade). Whereas the letter Z indicates variables that are at the city level includes the market concentration (hii) and urban development (ud).

The symbols "i" and "t" correspond to the terms "firm" and "year". The variables year_t,_ and θ_j_ represent a fixed effects specific to year and industry.

The study further developed a spatial model to analyze the nonlinear relationship between ESG and DVARE. An enhanced data quality, accuracy, and reliability is achieved by using a spatial model to gather and analyses spatial data related to ESG variables. It identifies areas of concentrated ESG risk, establish risk mitigation measures, and produce more comprehensive and accurate ESG reports.


$${DVARE}_{it}=\sum_{J=1}^{N}\rho W_{ij}DVA{RE}_{jt}+\beta_{1}ESG+\sum_{J=1}^{N}\beta_{2}W_{ij}ESG_{ij}+\beta_{3}X+\beta_{4}Z+\theta_{j}+\gamma_{t}+\varepsilon_{it}$$ (2)

The space weight matrix, denoted as W_ij_ in the study, is defined as the inverse geographic distance matrix. The variable θ_j_ represents the city fixed effects, which remain constant throughout different years. The variable γ_t_ represents the time fixed impact inside a specific year, which remains constant across different cities. The variables *ε_it_* represent random error terms.

### 3.2 Data

The researchers selected a sample period spanning from 2008 to 2019, taking into account the limitations imposed by the availability of Chinese customs data. This study employs two primary data sources, namely trade-level data and organizational-level data. The ASIE (Annual Survey of Industrial Enterprise) database is utilized to obtain data at the organizational level. The CCTS (Chinese Customs Trade Statistics) database provided the trade-level data. According to the detailed instructions provided by Scholars, the enterprise code, area code and phone number combination, industry code, industry year of establishment, main product code, and other factors were used to compare the annual aggregated CCTS database and ASIE database [43–45].

In light of the presence of outliers and noise within the matching sample, it is imperative to perform data cleansing before undertaking empirical Analysis. This procedure aligns with the methodology proposed by Feenstra [46]. The following criteria are applied to the data cleansing process: (1) Firms that fail to adhere to the Generally Accepted Accounting Principles (GAAP) are excluded from our Analysis. This includes instances where total assets exhibit negative values or are lower than net fixed or liquid assets, as well as cases where total liabilities are lower than liquid liabilities. (2) Exclusions are made for observations that lack essential data, including fixed assets, total assets, and industrial value-added. Additionally, firms with a gross value of output below 5 million are also removed. Finally, the study has 5447 effective annual observations from the years.

### 3.3. Variable measure

#### 3.3.1. Measurement of corporate ESG

The research conducted an extensive analysis to develop a complete ESG index that encompasses three key dimensions: environment, social, and governance. The index was employed to assess the ESG performance of various companies.

Within the framework of the social aspect, this study examines a set of variables that include safety production policy, creditor, consumer and employee protection policy, supplier protection policy and the presence of a shareholder protection policy. In addition, it will contribute towards the construction and enhancement of social responsibility, as well as system engagement in public relations and social welfare initiatives.

In the context of the environmental dimension, we have identified the subsequent variables: compliance of pollutant emissions with established standards, records of environmental petition incidents, presence of any environmental violations, and implementation of an operational environmental management system and attainment of ISO9001 certification for an environmental management system.

In the realm of governance, we have identified the subsequent variables: the alignment of the general manager and chairperson roles, the characteristics of the dominant shareholder, the company’s shares possessed by its ten largest shareholders, and the percentage of directors and supervisors’ shares compared to the total shares.

#### 3.3.2. Analysis of the PCA method

In accordance with the study of Qinglan Wu the study conducted a principal component analysis (PCA) to create a new ESG index to improve construct validity [34]. Principal Component Analysis (PCA) is a statistical technique used to analyse the underlying structure of a dataset composed of several variables. By using a PC statistical approach, it is possible to narrow down the amount of information in the dataset and discover relationships between individual elements. The weights assigned to each factor in the linear composite of the original variables correspond to the eigenvectors of the correlation within the covariance matrix. These weights aim to maximise the amount of variance accounted for by each subsequent factor in the original dataset. PCA iterates to extract principle components until a substantial portion of the variation is accounted for. The study performed the Bartlett test and KMO test on the dataset to assess the suitability of the variables for PCA. Variable correlation is measured by the KMO test. The degree of commonality across variables increases with increasing KMO value. The sphericity test’s p-value corresponds to the Bartlett test value. Correlation between the variables is inferred when the p-value is less than 0.05; however, KMO values for all dimensions exceed 0.5, according to results in Table 1. It indicates that our data is subject to PCA.

**Table 1:** PCA suitability test.

Variable	Test	Value
**S**	P value	0.001
	KMO	0.717
	Degree of freedom	27
**E**	P value	0.000
	KMO	0.773
	Degree of freedom	31
**G**	P value	0.000
	KMO	0.801
	Degree of freedom	38

In PCA, eigenvalues greater than one are the standard principle components. Table 2 shows that there are three main components that make up the environmental dimension. The combined contribution of the three primary components amounts to 75,6%, surpassing the threshold of 50%. In the context of the social dimension, it is observed that there are three primary components. The combined contribution of the three primary components amounts to 80.7%. The number of primary components under the corporate governance dimension is 2. The combined contribution of the two primary components amounts to 67.3%. The findings of the main component analysis are satisfactory.

**Table 2:** Factor contribution and eigenvalue.

Variable	Component	Proportion	Eigenvalue	Cumulative
E	C1	0.401	1.403	0.401
	C2	0.192	1.142	0.593
	C3	0.163	1.112	0.756
	C4	0.123	0.575	0.879
	C5	0.121	0.433	1.000
S	C1	0.431	1.677	0.431
	C2	0.210	1.221	0.641
	C3	0.166	1.033	0.807
	C4	0.117	0.325	0.924
	C5	0.055	0.223	0.979
	C6	0.021	0.162	1.000
G	C1	0.412	1.475	0.312
	C2	0.261	1.333	0.673
	C3	0.215	0.127	0.888
	C4	0.112	0.124	1.000

The environmental, social, and governance scores were calculated independently using the following formulas:

E_score = (0.401 * Comp1 + 0.192* Comp2 + 0.163* Comp3)/ (0.401+ 0.192 + 0.163)

S_scores = (0.431 * Comp1 + 0.210 * Comp2 + Comp * 0.166)/(0.431 + 0.210 + 0.166)

G_score = (0.412 * Comp1 + 0.261* Comp2)/ (0.412 + 0.216)

The corporate ESG is ultimately established by calculating the average weights assigned to each of these three dimensions.

### 3.3.3. Measurement of the DVARE

According to Johnson and Noguera, DVARE is the proportion of domestic value added to exports [15]. According to Upward et al., we used a mathematical method with firm-transaction-level data to compute DVARE [1].


$$DVARE=\left(\frac{Y^{P}}{Y}\right)\left(1-\frac{N^{P}}{Y^{P}}\right)+\left(\frac{Y^{o}}{Y}\right)\left[1-\frac{N^{o}}{\left(C+Y^{O}\right)}\right]$$ (3)

The import intermediates employed in ordinary and processing trade are referred to as "N^O^" and "N^P^," respectively. Here, superscript O symbolizes ordinary trade and P processing trade. However, the enterprise’s overall production consists of Y^P,^ Y^O,^ and C. The term "(C+Y^0^)" refers to the sum of ordinary exports and product sales in the domestic market. However, Eq (3) yields an inflated calculation of the domestic content in exports, in contrast to the findings derived from sector-level estimations that rely on the I-O table. Following the study of Sun et al., certain adjustments were made to the study in order to achieve more precise measurement results [17]. The study first examines and revises firms’ imports using Eq (4) to calculate N^P^adj and N^O^adj, which signify the imports engage in processing trade and ordinary trade. These initiatives are unique in nature and supplement Hu’s evaluation of DVAR [47] by not only removing the intermediary firms but also by making necessary revisions to the initial biased N^P^ and N^O^.


$$N_{adj}=\frac{N}{1-ratio}$$ (4)

In addition, the foreign component of DVAR in export is further identified and eliminated in this work, which Hu, (2021) neglected in their Analysis and repressed the formula for DVARE based on the study of Sun et al. (2023). This research identifies the BEC codes for intermediates using a comprehensive description of the Broad Economic Categories (BEC). Additionally, the N^O^_adj_ is transformed into N^O^_adj_BEC_. Following the aforementioned procedures, the DVARE computation method is re-expressed as

$$DVARE=\left(\frac{Y}{Y}\right)\left(1-\frac{N_{adj}^{p}}{Y^{p}}\right)+(\frac{Y_{0}}{Y})\left\{1-\frac{\left[N_{adj_{BEC}}^{o}+\varphi (N^{T}-N-N_{adj_{BEC}}^{o})\right]}{\left(C+Y^{O}\right)}=\right\}$$ (5)

N^T^ stands for the total enterprise input of intermediate factors. We exclude excessive import and export enterprises from the sample by referring to Kee & Kong [48].

### 3.4. Technological turbulent

In line with David Bendig’s research, we measured moderator technical turbulence (technological rate of advancement within an enterprise) by comparing the R&D investments made by businesses to their sales employing data from the CSMAR and Wind databases [49]. Descriptive statistic for all variable are displayed in Table 3.

**Table 3:** Summary statistic.

Variable	Description	N	Mean	SD	minimum	maximum
DVARE	value added rate of export (%)	5929	15.24139	3.922483	0	20.25133
ESG	Environmental, social, and governance	5,929	6.612582	1.036212	2	9
Technological turbulence	the rate at which the industry adopts new technologies	5,929	.3558242	.1773747 .	0.005	.867813
Age	ln(age of firm +1)	5,929	2.188782	.7161005	.693147	.693147
UI	Average industry involvement in global value chains as determined by the World InputOutput Database (WIOD).	5,929	.4173838	0.2318	.001579	.872362
foe	if it is a Sino-Foreign Cooperative, Joint Venture, or 100% Foreign-Owned Business, foe = 1; otherwise, foe = 0	5,929 .	.2125148	.4091216	0	1
exd	Export delivery value/business sale	5,929	58.33969	1.560445	9.5174	74.8946
lev	the proportion of all liabilities to all assets at year’s end.	5,929	.2487141 .	1.1638552 .	.001594	.712862
trade	If the business processes trade, trade = 1; otherwise, trade = 0.	5,929	.4169337	4930933	0	1
exit	If the firm leaves in the coming year, exit = 1; otherwise, exit = 0.	5,929	.2349469	.4240014	0	1
hii	The entire market value of the industry multiplied by the square of each company’s market share	5,929	2.175816	.1922154	1.609438	2.70805
ud	ln(Urban GDP per capita)	5,929 .	.4603485	.2073222 .	.064671	.894039

## 4. Empirical analysis and discussions

### 4.1. Baseline regression

Eq (1) was used to compute the benchmark results, which are shown in Table 4, whereas columns (1) through (4) gradually incorporate the city and firm-level control variables. Colum (2) and (3) display the estimated findings with specific control factors, whereas Column 1 displays the estimation values without any control variables. After considering the control variable, industry, and year-fixed effects, the ESG coefficient is determined to be 0.3894, which is significant at 1%. These findings indicate that for each additional ESG unit, a firm’s DVAR increases by an average of 0.3894 units. Furthermore, it can be noted that a significant number of the coefficients related to the control variables demonstrate statistical significance. The results of this research imply that raising ESG standards boosts exports’ DVAR. As a result, Hypothesis 1 is supported. Businesses that implement effective ESG practices can enhance the exports’ DVARE.

**Table 4:** Baseline regression.

	DVARE	DVARE	DVARE	DVARE
ESG	0.2376***	0.2308***	0.2161***	0.3894***
	(4.7173)	(4.5481)	(4.2513)	(8.1454)
age		-0.7438***	-0.8849***	-0.7558***
		(-9.8638)	(-10.9486)	(-9.6231)
ui		1.6584***	1.5120***	1.4579***
		(6.7266)	(6.0792)	(6.2105)
exd		-0.0070	-0.0075	0.0003
		(-1.7283)	(-1.8586)	(0.0845)
lev		3.2407***	3.0500***	2.0524***
		(8.0788)	(7.5077)	(5.2107)
foe		0.1701	0.2392*	-0.0020
		(1.5014)	(2.1155)	(-0.0183)
trade		-0.0536	-0.0586	0.1284
		(-0.5115)	(-0.5630)	(1.3449)
exit		0.3635**	0.2936*	0.2566*
		(2.8020)	(2.2646)	(2.0986)
ud			1.6885***	2.7392***
			(5.5776)	(9.1881)
hii			0.4002	0.6932**
			(1.4203)	(2.5803)
_cons	13.6701***	14.0029***	12.8944***	10.9817***
	(41.3632)	(37.3907)	(18.8124)	(15.8611)
F	22.2530	20.8659	20.9785	17.5152
r2_a	0.0038	0.0425	0.0497	0.1608
N	5.9e+03	5.9e+03	5.9e+03	5.9e+03
ind				yes
year				yes

The t statistics are enclosed in brackets. A statistically significant value at the 1%, 5%, and 10% levels is indicated by the symbols ***, **, and *.

### 4.2. Spatial panel model selection

A set of predetermined screening guidelines must be followed in order to choose an appropriate spatial panel model. The models in this research are filtered using the criteria outlined by Elhorst [50]. Initially, the study performed OLS regression; subsequently, the LM test was run based on this. Then, LM and Robust LM statistics are used to filter the SAR or SEM. When just LM-Lag statistics are significant, the SAR model is chosen. All four statistics in Table 5 are significant, suggesting that further research calls for the use of the spatial econometric model.

**Table 5:** Model selection.

	OLS		SEM	SAC	SAR	SDM
	FE NF					
Lag-LM	216.441*** (0.000)	240.328***(0.000)				
Error-LM	202.631*** (0.000)	214.220*** (0.000)				
Error Lag-LM	1.495** (0.027)	3.201**(0.014)				
Robust Lag-LM	6.232*** (0.003)	9.110*** (0.001)				
LR test			2.11(0.014)			
Wald test					3.10(0,069)	
AIC				3221.11		3306.31
BIC				3289.28		3399.18

The statistics for the Wald and LR test χ2 are shown. and square brackets encompass the relevant *P* -value.

Furthermore, assuming the selection of SDM, the Wald or LR test is conducted to evaluate the fitness of the SDM. LR test and Wald test both produced statistically significant outcomes; however, it is important to highlight that the AIC and BIC values corresponding to the SDM (3306.31 and 3399.18), respectively surpass the SAC (3221.11 and 3289.28, respectively). This discrepancy suggests that the configuration of the SDM is superior to that of the SAC.

#### 4.2.1. Spatial econometric regression

Table 6 displays the findings of the study of the spatial effect estimation. Columns (1)–(3) display the estimation results for SAR, SAC, and SEM. Column 4 reports the estimated outcomes of SDM. The spatial term coefficients for all three types of models show a positive trend, indicating that the local DVARE will be impacted by the activities of the DVARE in other provinces. A statistically significant correlation has been found between ESG performance and the local and surrounding DVARE, as indicated by the positive coefficients of *ER* and W*ER. Explanation illustrate that domestic intermediate inputs incur higher costs than their foreign counterparts do, and there is no capacity to improve their quality during relatively relaxed environmental regulations [18]. Domestic businesses increased their purchases of domestic intermediate goods by implementing technical advancements to lower pollution emissions, increase production efficiency, and improve product quality.

**Table 6:** Spatial effect estimation results.

Variable	SAR	SAC	SEM	SDM
ESG	0.2807***(3.5737)	0.4791**(2.0979)	0.3340**(2.8810)	0.3325***(3.1530)
W*ESG				0.7751*** (6.0991)
Industry FE	Added	Added	Added	Added
Year FE	Added	Added	Added	Added
Control V	Added	Added	Added	Added
R^2^	0.1029	0.3311	0.3361	0.421
Observation	5930	5930	5930	5930

In relation to the spillover phenomenon, when the movement of capital between the local province and its surrounding province occurs without incurring any costs, the marginal production of capital in both the local province and its surrounding provinces is equal, assuming they are subject to the same level of ESG practices. The implementation of local ESG practices might result in a rise in production costs within the region, consequently resulting in a reduction in the marginal product of capital [34], and assuming that the expenses related to the circulation of products and amenities within local and nearby provinces are lower than the supplementary costs incurred due to adopting high ESG. In this scenario, it is possible for local producers to expand their operations into nearby provinces, influencing the economic value of these provinces.

### 4.3 Robustness check

Several tests for robustness were run on the results of the benchmark regression. First, we took firm entry or exit as a potential source of bias in the predicted results. Since n≤2, is too small, and n≥6 is large to provide a reliable result therefore in line with the study of Qinglan et al, we kept a sample of businesses that had been around for at least three, four, and five years to derive a reliable result [34]. during the sample period and reran the Eq (1). The coefficients of ESG in Table **7** are 0.122, 0.105, and 0.092, respectively. These coefficients exhibit a modest decrease compared to the ESG coefficient seen in the benchmark regression. However, their statistical significance is maintained at the 1% level. Also, the results demonstrate that firms’ entrance or leave had no impact on the benchmark regression, and it accurately represents the influence of ESG performance on export DVAR.

**Table 7:** Excluding corporate exits and entrances effect.

	n≥3	n≥4	n≥5
ESG	0.122***	0.105***	0.092***
	(5.229)	(8.003)	(9.114)
Control variables	Added	Added	Added
Year FE	Added	Added	Added
Industry FE	Added	Added	Added
N	5930	5930	5930
Adj R^2^	0.089	0.089	0.091

The t statistics are enclosed in brackets. A statistically significant value at the 1%, 5%, and 10% levels is indicated by the symbols ***, **, and *.

The study uses domestic value-added share (DVS) instead of DVAR, based on a study by Upward et al. [1], to assure the reliability and validity of the results. The ESG coefficient maintains statistical significance as observed in Table 8, Column (1). This means that changing the primary indicators of domestic export value-added does not affect the basic conclusion.

**Table 8:** High dimensional fixed effect and alternative dependent variable.

	DVS	DVARE	DVARE	DVARE	DVARE
ESG	0.0045***	0.190**	0.191**	0.162***	0.175***
CV	Added	Added	Added	Added	Added
Y FE	Added	Added	Added	Added	Added
P FE	Added	Added	Added	Added	Added
I FE	Added	Added	Added	Added	Added
P*Y FE	N(No)	N	Added	N	Added
I*Y FE	N	N	N	Added	Added
Adjusted R^2^	0.1152	0.0671	0.0132	0.0123	0.0334

Third, to lessen the effect of unobservable elements, the study added high-dimensional fixed effects to Eq (1). Column (2) in Table 9 explicitly regulates industry(I) and year(Y) fixed effects at the province(P) level. Column (3) incorporates year fixed effects × province to account for variables unique to each province that may vary with time. Industry-specific characteristics that vary within time are further reflected by the addition of year fixed effects ×industry in column (4). Column (5) presents the fixed effects for year × province and industry × year, as derived from the benchmark. According to the results, improving a company’s ESG performance is associated with a higher rate of domestic value-added exports. The acquired result aligns with the benchmark regression, confirming the strength and reliability of the empirical findings in this research.

**Table 9:** Results for instrumental variables using the 2SLS method.

First stage			Second stage	
	ESG	ESG	DVARE	DVARE
ESG			0.033** (2.118)	0.021** (3.0091)
I_ESG1	0.778*** (4.4545)			
P_ESG1		0.711*** (6.229)		
Control Variables	Added	Added	Added	Added
Industry FE	Added	Added	Added	Added
Year FE	Added	Added	Added	Added
Observation	Added	Added	Added	Added
F value	3.72***	4.011***	15.92***	16.20***
Adj R^2^	0.0472	0.0491	0.0362	0.0393
Robust F statistic (Weak instruments test)	17.039***	18.044***		

### 4.4. Endogeneity test

Instrumental variables were included to address endogeneity concerns throughout the estimation process. The primary issue of endogeneity encountered with the current research pertained to the phenomenon of inverse causality. One potential explanation is that companies with a high level of export DVAR own more capital; this increased export DVAR intensity further contributes to the improvement of an enterprise’s ESG performance. Also, the growing focus that overseas markets place on corporate social responsibility (CSR) serves as a key driver for companies looking to enhance their ESG performance in these countries. Hence, it is imperative that the instrumental variables meet the requirements of correlation and endogeneity.

The instrumental variables primarily consist of the same province average ESG eliminating this firm(P-ESGI), and the industry average ESG eliminating this firm (I_ESG1). The first-stage regression findings of the 2SLS estimation, presented in Table 9, indicate that at 1% significance, the instrument variables’ coefficients show a positive connection with the ESG. Both instrumental variables have successfully passed the weak identification test, as indicated by their statistically significant F values. The available evidence supports the conclusion that a weak instrumental variable does not exist. The presence of statistically significant coefficients for ESG at 5% levels in the second phase suggests a favorable correlation between ESG and DVARE. This leads to the strong conclusion that ESG practices raise the firm’s DVARE.

### 4.5. Analysis of heterogeneity

#### 4.5.1. Firm heterogeneity

Export DVAR can be considerably increased by achieving strong ESG performance, as the above results show. Does the impact of ESG practices on export DVAR vary between enterprises with various characteristics? This study takes into account potential differences in the effects of ESG performance on DVARE by classifying the firms as either large (LAEs) or small and medium-sized (SMEs). The statistical significance of the ESG coefficient (0.3310) for LAEs is determined at the 1% level, as indicated in Table 10.; however, the ESG coefficient for SMEs did not have a statistically significant effect. According to the result, the positive relationship between ESG performance and export DVAR, which was seen in the benchmark regression, is largely attributable to the LAEs in the sample.

**Table 10:** Firm heterogeneity analysis.

	OT	MT	PT	SMEs	LAEs
ESG	0.1810	0.1408**	0.2012***	0.2191	0.3310***
	(0.112)	(2.021)	(5.003)	(0.104)	(4.010)
Control variables	Yes(Y)	Y	Y	Y	Y
Firm fixed effects	Y	Y	Y	Y	Y
Year fixed effects	Y	Y	Y	Y	Y
Constant	1.1601***	1.753***	0.8831***	1.5521**	0.7325***
adj R^2^	0.0441	0.1130	0.0530	0.1290	0.0442
Observations	2075	2102	1723	2687	3213

The t statistics are enclosed in brackets. A statistically significant value at the 1%, 5%, and 10% levels is indicated by the symbols ***, **, and *.

This study divides export companies into three distinct categories based on the CCTS database’s trade patterns: ordinary trade (OT), mixed trade (MT), and processing trade (PT). The findings are presented in columns 1 to 3 of Table 10. The statistical insignificance of the ESG coefficient in the ordinary trade sample suggests that it does not exert a substantial influence on the final result. However, it is evident that the implementation of ESG practices has led to notable enhancements in DVARE within the context of processing and mixed trading patterns.

#### 4.5.2. Regional heterogeneity

Over 90% of the firms in the sample come from the eastern regions. Less than 10% of firms are from central and western regions. It fits with what was already known, that 90% of all Chinese exports come from the eastern part of the country. The findings in Table 11 indicate that the implementation of ESG practices positively affects the DVARE of the eastern region. The observed phenomenon can be attributed to the East region’s substantial increase in exports and its advanced stage of economic development [17]. Conversely, because the economies and export sectors of the western and central regions are underdeveloped, policy efficacy in these two sectors is negligible since there is little incentive to boost the DVARE.

**Table 11:** Regional heterogeneity analysis.

	Western	Central	Eastern
ESG	0.0912	0.1611	0.2047***
	(0.114)	(0.161)	(5.004)
Constant	1.6610***	1.0991**	1.2230***
Firm FE	Added	Added	Added
Year FE	Added	Added	Added
Control variables	Added	Added	Added
Adj R^2^	0.6621	0.5910	0.7120
Observation	433	784	4713

### 4.6. Mechanism analysis

In order to determine the presence of a moderating effect, a moderating model is developed that focuses on how technology turbulence affected the connection between DVARE and ESG practices.


$$DVARE_{it}=\gamma_{0}+\gamma_{1}ESG_{it}+\gamma_{2}ESG_{it}*M_{it}+\gamma_{3}M_{it}+\gamma_{4}X+\gamma_{5}Z+\theta_{j}+\gamma_{t}+\varepsilon_{it}$$ 6

The "M_it_" denotes the moderating variables. The remaining variables are identical to those of the benchmark settings. The term of interest is the interaction term of ESG_it_.* M_it,_ as shown in Eq (6). The coefficient γ_2_ indicates how the technological change of the businesses may moderate the impact of ESG policies. Table 12 contains the outcomes of the estimations for Eq (6). The results demonstrate that the coefficient of ESG_it_*M_it_ is statistically significant and positive in Column (2).

**Table 12:** Mechanism analysis.

Variable	DVAR	DVAR
ESG	0.3894***(8.1454)	0.5864***(4.8240)
Technological turbulence*ESG		9.2394***(4.6527)
Technological turbulence		-0.8809**(-2.9531)
Age	-0.7558***(-9.6231)	-0.7061***(-9.1733)
ui	1.4579***(6.2105)	0.7022**(2.9464)
exd	0.0003(0.0845)	-0.0003(-0.0796)
lev	2.0524***(5.2107)	2.1727***(5.5053)
foe	-0.0020(-0.0183)	-0.0185(-0.1728)
trade	0.1284(1.3449)	0.0926(0.9818)
exit	0.2566*(2.0986)	0.1715(1.4125)
ud	2.7392***(9.1881)	3.0367***(10.3058)
hii	0.6932**(2.5803)	0.4794(1.8230)
Industry	Yes	Yes
Year	Yes	Yes
Constant	10.9817***	9.1515***
Observation	5.9e+03	5.9e+03
Adj R^2^	0.1608	0.1799

The t statistics are enclosed in brackets. A statistically significant value at the 1%, 5%, and 10% levels is indicated by the symbols ***, **, and *.

This suggests that a higher level of technological change improves the favorable effect of ESG practices on the exports’ DVAR. Further, in order to examine the interaction effect, we calculated the correlation between ESG practices and DVARE at different levels of technological turbulence.

The three levels were determined as follows: low (mean—1 (SD)), high(mean + 1(SD) standard deviation), and mean technological turbulence(mean). We performed a slope test to see if the various technological turbulence levels differed considerably from zero. Table 13 shows that each of the three degrees of technological turbulence has significant slopes at 5% and 1%, which provides statistical evidence for the third hypothesis (H3) of a moderated connection. Explanation illustrate that utilizing modern technologies that provide transparent, real-time, organized, and authenticated data can assist address the lack of accessible ESG data to some extent. Businesses that operate in highly technologically advanced environments will be more adept at putting their ESG policies into practice, which will raise export DVAR.

**Table 13:** DVAR at three different levels of technological turbulence.

	ESG(low)	ESG(mean)	ESG(high)
DVARE			
technological turbulence slope = 0.005	5.29**	5.36**	5.13**
technological turbulence slope = 0.033	8.33***	8.41***	8.07***
technological turbulence slope = 0.061	11.09***	11.15***	10.92***

## 5. Conclusion and recommendation

The study looks into how ESG performance relates to the domestic value-added rate of export using a well-established ESG metric of Chinese listed companies. The following is a summary of the study’s findings. (1) The adoption of ESG practices has led to a significant increase in the exports’ DVAR. (2) The implementation of ESG practices has been observed to impact the DVARE of local areas directly. Additionally, because of the spatial spillover effect, these practices significantly impact the DVARE of nearby provinces. (3) The major areas where ESG practices are seen to have an impact on the DVARE are in the large, mixed, and processing trade organization as well as enterprises located in the eastern region. (4) Analysis of the underlying mechanisms reveals that the technology turbulence enhances the favorable influence of ESG performance on Chinese enterprises’ DVARE.

The paper’s findings lead us to the following policy recommendations:

In order to expand trade and ESG policies in the future, it will be necessary to gradually increase the adoption of "front-end prevention" measures. As the study concludes, ESG practices increase companies’ DVARE. The progressive acceleration of the adoption of such ESG practices can help businesses become more competitive, expedite the green transformation, and fulfill their social responsibility, all of which will help Chinese enterprises enhance DVARE and ultimately help Chinese corporations maintain sustainable development.

ESG practices have a significant relationship with exports’ DVAR through the spatial spillover effect. It might affect economic activity, especially when it comes to urban exports, leading to a time characterized by significant challenges and difficulties. Hence, it is imperative for the government and various sectors of society to enhance the ongoing implementation of ESG policies. This necessitates the coordination of policies across multiple domains, including finance and taxation, foreign trade, and innovation [44].

The results of the heterogeneity test demonstrate the disparities in the rates of development of China’s regions and organizations, as well as the limitations of a single ESG framework in terms of facilitating the growth of all kinds of organizations and areas. Consequently, governments must carefully consider ESG policies, enhance the disclosure of ESG information, and provide financial assistance to SMEs, ordinary trade organizations, and Western and middle regions so they can advance ESG practices.

Businesses in an era of rapid technological development face great pressures to adapt. We suggest that by utilizing advanced technologies, an enterprise might take advantage of enhancing its ESG policies. A previous study findings show that 53% of participants in the BlackRock study expressed concern regarding the absence of accessible ESG data, which is necessary to evaluate the enterprise’s sustainability. The adoption of advanced technologies, which offer transparent, real-time, organized, and data authentication, will help to partially tackle this issue [18]. Thus, our study proposes that organizations should persist in expanding their investment in the use of innovative technologies to improve their ESG performance and foster corporate DVARE.

## Supporting information

S1 Data (XLSX)
